# Rhinology, Laryngology and Otology, and Their Significance in General Medicine

**Published:** 1901-02

**Authors:** 


RHINOLOGY, LARYNGOLOGY AND OTOLOGY, AND THEIR SIGNIFICANCE IN GENERAL MEDICINE. By E. P. FRIEDRICH, M. D., Privat docent at the University of Leipzig. Authorized translation from the German. Edited by H. HOLBROOK CURTIS, M. D., Consulting Surgeon to the New York Nose and Throat Hospital and to the Scarlet Fever and Diphtheria Hospitals. Octavo, pp. 348. Philadelphia and London: W. B. Saunders & Co. 1900. [Price, $2.50.]

Within the last few years so many books have been written upon the subjects of rhinology, laryngology and otology, that it is refreshing to find one entirely unlike any of the others, and just such a work is Dr. Friedrich's book. Nothing similar to it has been written on these subjects up to the time of its publication in either the English, German, French or Italian languages, although Mauritz Schmidt's Diseases of the Upper-air Passages, and some other works on kindred subjects have been written, considering the relation of such diseases to general medicine.

This is a book by a man familiar with the pathology and symptoms of general diseases, as well as by one who is an expert in the diagnosis and treatment of diseases of the nose, throat and ear. This work, instead of being a compilation, is a union of the author's large experience in the clinic at Leipzig with that of his fellow workers in his specialty in Germany, Austria, England, Italy, France and America; then experiences he found recorded in such records as Haymann's Handbook, Frankil's Archiv für Laryngologie, The Archives of

Otology, Lemon's Centralblatt für Laryngologie, The Journal of Laryngology, as well as in many recent monographs and text-books.

The work is rich in material, every sentence is the statement of an established clinical fact verified in the post mortem room, or in the laboratory, which makes a detailed review of the work impossible. Diseases of the nose, throat and ear are considered in their relation to each other and in their relation to (1) diseases of the rest of the respiratory tract; (2) diseases of the circulatory system; (3) diseases of the digestive tract; (4) diseases of the blood; (5) chronic constitutional diseases; (6) acute infectious diseases; (7) chronic infectious diseases; (8) diseases of the kidneys; (9) diseases of the skin and of the sexual organs; (10) diseases of the eye; (11) intoxications; (12) nervous diseases. In an appendix, nasal reflex neuroses, the significance of some of the cranial nerves in rhinology and otology, and diseases of the meninges and of the cerebral sinuses and their significance in connection with the nose, larynx and ears, are all ably and exhaustively dealt with.

Such an enumeration serves to explain how complete this book is. The chapters treating of nervous diseases are interesting and rich in new material, as are also those upon the exanthemata and their sequels. Dr. Curtis, the editor, is to be commended for presenting to English readers this valuable book.

The translation has been well done, for the translator has not only succeeded in presenting the author's meaning, but has also presented to us the text in natural English prose.

Messrs. W. B. Saunders & Company are to be congratuated not only upon the style of this book, but also for having presented to medical readers in this country the valuable Hand-Atlases, which are among the best of the medical books which have of recent years been translated from the German into English. W. S. R.

